# Factors Associated With Opioid Agonist Treatment Engagement and Harm Reduction Service Use: Findings From the New South Wales Opioid Dependence Survey

**DOI:** 10.1111/dar.70132

**Published:** 2026-03-18

**Authors:** Thomas Santo, Chrianna Bharat, Craig Rodgers, Mary Harrod, Sophia Taylor, Emma Zahra, Rachel Sutherland, Amy Peacock, Jason Grebely, Matthew Hickman, Michael Farrell, Louisa Degenhardt

**Affiliations:** ^1^ National Drug and Alcohol Research Centre UNSW Sydney Sydney Australia; ^2^ Alcohol and Drug Services, St Vincent's Hospital Sydney Australia; ^3^ The NSW Users and AIDS Association Sydney Australia; ^4^ School of Psychology University of Tasmania Hobart Australia; ^5^ The Kirby Institute UNSW Sydney Sydney Australia; ^6^ Population Health Science, Bristol Medical School University of Bristol Bristol UK

**Keywords:** incarceration, mental disorders, opioid agonist treatment, opioid dependence

## Abstract

**Introduction:**

Opioid agonist treatment (OAT) is one of the most effective treatments for opioid dependence, but there can be barriers to accessing treatment. This study examined characteristics associated with OAT engagement—never, previous or current—among people with opioid dependence in NSW, Australia, following recent reforms to OAT access.

**Methods:**

Between October 2023 and March 2024, people with opioid dependence were recruited from services and community settings across NSW. Participants completed structured interviews on socio‐demographics, mental health disorders, substance use and use of prevention and treatment services. Logistic regression was used to examine associations between participant characteristics and engagement with OAT.

**Results:**

Of 403 participants (mean age 44; 65% male), 77% were currently receiving OAT (60% methadone, 11% sublingual buprenorphine and 27% long‐acting injectable buprenorphine), 13% previously and 9% never. Differences between OAT status groups included those currently receiving OAT reporting lower rates of homelessness and extra‐medical opioid use, and greater group therapy engagement and past‐year receipt of naloxone kits compared to those previously and never receiving OAT. Past‐month daily injecting drug use was higher among those previously and never receiving OAT; those previously receiving OAT also reported the highest rate of past‐year supervised injecting facility use and overdoses in the past 6 months.

**Discussion and Conclusions:**

Those not receiving OAT reported greater social instability, recent injecting drug use and drug‐related harm. Variation in harm reduction service use suggests tailored strategies are needed to reach those outside OAT. These findings highlight the need for integrated, diverse strategies to address dependence‐related harms.

## Key Point

- A cross‐sectional survey was conducted in New South Wales (October 2023–March 2024) with 403 people with opioid dependence recruited via needle and syringe programs, treatment services, pharmacies and online.
- Socio‐demographic, mental health and substance use characteristics are summarised overall and by opioid agonist treatment (OAT) status, comparing people currently (77% of cohort), previously (13%) and never (9%) receiving OAT.
- Compared to those currently receiving OAT, people not receiving OAT (never or previously) were more likely to be homeless, inject daily and use opioids extra‐medically (e.g., heroin, buprenorphine).
- Harm reduction service use was high overall, with higher naloxone uptake among those in OAT than not in OAT (59% vs. 32%), and greater use of supervised injecting facilities among those never in OAT than currently in OAT (29% vs. 22%).

## 1 Introduction

Opioid dependence is a major public health issue that affects more than 40 million people worldwide [1]. Opioid dependence is characterised by a cluster of cognitive, behavioural and physiological symptoms resulting in continued opioid use despite significant harms, and is associated with various physical and mental health disorders [2], including blood‐borne viral infections [3], self‐harm and suicide [4], non‐fatal and fatal overdose [1] and all‐cause mortality [5]. Australia has a relatively high rate of opioid dependence‐related harm [6]. The social costs of extra‐medical opioid use in Australia in 2015–216 have been estimated at $15.67 billion [7]. There were 1073 opioid‐induced deaths among Australians in 2020, including more than 500 in New South Wales (NSW) alone [8].

Opioid agonist treatment (OAT) with methadone or buprenorphine, both designated as World Health Organization Essential Medicines [9], is the most effective evidence‐based treatment for opioid dependence. OAT substantially reduces withdrawal symptoms, craving and ultimately reduces illicit opioid use [1], injecting‐related harms [10] and mortality [11]. The benefits of OAT can be further supported through linkage to additional services, including harm reduction interventions (such as needle and syringe programs [NSP] and naloxone provision), mental health care and social support services [12, 13, 14].

In Australia, access to methadone and buprenorphine for OAT is subsidised by the Australian Government. National estimates indicate that OAT coverage among people with opioid dependence in Australia meets, and in some regions exceeds, World Health Organization targets for high coverage [15]. However, maintaining engagement in OAT remains difficult and long‐standing inequities in access and service delivery continue. The 2022 post‐market review of the Opioid Dependence Treatment Program highlighted substantial shortfalls in rural and remote service provision [16], as well as the considerable financial burden to clients being dispensed OAT in the community setting [17, 18].

Recent developments in the treatment of opioid dependence in Australia, including the introduction of long‐acting injectable formulations of buprenorphine in 2019 and major decreases in out‐of‐pocket dispensing fees in 2023 [19], were aimed at improving access to OAT [20]. These changes in service delivery may have reduced or eliminated barriers to accessing and continuing OAT. Accordingly, a contemporary analysis of the characteristics of individuals with opioid dependence, alongside their patterns of OAT engagement, is warranted to better understand the current treatment landscape. Investigations by OAT medication type could also provide insights regarding integration of the new formulation. Importantly, such analyses should move beyond treatment‐only data sources [21], which typically lack information on critical contextual factors. For example, socioeconomic factors, polysubstance use and injection related risk behaviours, engagement with harm reduction and social support services often cannot be determined from clinical data alone. Exploring patterns of substance use and harm reduction engagement among individuals who are not engaged in OAT may provide further insights into the treatment gap and identify opportunities for improving the reach and responsiveness of services.

Therefore, the aims of this study were to:

1. Characterise socio‐demographic, mental health and substance use characteristics of a sample of people who are opioid dependent, and examine whether these are associated with current or past receipt of OAT.
2. Summarise the characteristics of OAT provision, according to medication type.
3. Describe the use of other harm reduction and support strategies, according to current OAT status.

## 2 Methods

### 2.1 Study Design

The NSW Opioid Dependence Survey is a cross‐sectional study of adults (≥ 18 years) living in NSW with current opioid dependence. Eligible participants had to meet the following criteria: (i) voluntarily give informed consent; (ii) be aged 18 years or older; (iii) reside in NSW; and (iv) have opioid dependence. Opioid dependence was defined as either having received OAT in the past month or having used non‐prescribed pharmaceutical opioids and/or heroin on at least 21 of the past 28 days. Participants were recruited between 17 October 2023 and 22 March 2024. Flyers were distributed across 12 local health districts in NSW, Australia through a variety of sources including peak consumer advocacy groups, OAT dosing sites and pharmacies, NSPs, Sydney's Medically Supervised Injecting Centre, web advertising, snowballing and other community organisations providing support for people who use drugs (see Acknowledgements).

The survey sample size was determined based on power calculations designed to detect significant differences in rare events. We used statistics from a recent survey of people with opioid dependence in NSW [22] to estimate the prevalence of participants who will be out of treatment and past‐year overdose among a current sample of people with opioid dependence in NSW. Overdose was selected as the primary outcome because it is clinically important yet relatively uncommon, providing a sensitive test of group differences. Power calculations, conducted with a 95% confidence level, a Type I error rate (*α*) of 0.05 and a Type II error rate (*β*) of 0.10, estimated a minimum sample size of 400 participants.

Eligible participants had to meet the following criteria: (i) voluntarily give informed consent; (ii) aged 18 years or older; (iii) reside in NSW; and (iv) have opioid dependence. Opioid dependence was defined as either having received OAT in the past month or having used non‐prescribed pharmaceutical opioids and/or heroin at least 21 of the past 28 days. Participants completed a structured interview, administered by one of the research team, either face‐to‐face or remotely via telephone or online video‐call. The survey took approximately one hour to complete, and participants were reimbursed $40 AUD for time and out‐of‐pocket expenses incurred.

### 2.2 Survey Development

The survey was developed by researchers with experience conducting epidemiological studies, including large‐scale community and clinical population surveys, focusing on people with a history of opioid dependence [22, 23, 24, 25, 26, 27], and included consultation with key stakeholders with in‐depth knowledge of opioid dependence, including clinicians and representatives from community organisations. The survey had 12 primary sections: socio‐demographic characteristics, substance use history, opioid dependence, OAT, treatment preferences for opioid dependence, quality of life, mental health disorders, adverse childhood experiences (ACE), harm reduction services, overdose history, incarceration and arrest history and physical health. An overview of the questions and tools administered in the survey is provided (Appendix A).

### 2.3 Measures

#### 2.3.1 Aim 1: To Characterise Socio‐Demographic, Mental Health and Substance Use Characteristics of the Sample

Demographic characteristics included age, gender, country of birth, education, employment, housing stability (including current and previous experiences of homelessness), and past‐year and lifetime incarceration. Homelessness was defined as primary, secondary and tertiary homelessness (including caravans, hostels, couch‐surfing, sleeping rough and insecure accommodation) [28].

Substance use history focused on lifetime and recent extra‐medical opioid use and other drugs of interest. Questions were adapted from the Australian Illicit Drug Reporting System, an annual national survey of people who inject drugs that has operated since 2000 [29]. For each major substance class, including heroin, non‐prescribed pharmaceutical opioids such as methadone, buprenorphine formulations, oxycodone, morphine, codeine, tramadol, tapentadol and fentanyl as well as benzodiazepines, methamphetamine or amphetamine and cocaine, we recorded whether the participant had ever used the substance. We also recorded when the substance was last used, which allowed us to classify use as current, within the past month or more than a month ago. For each of these substances, we recorded whether the participant had ever injected it and, where relevant, when injecting last occurred. Injecting drug use was assessed by asking participants whether they had ever injected any drug, their age at first injection, whether they had injected in the past 12 months and in the past month and, among those injecting in the past month, their average injecting frequency. Lifetime and past 6‐month non‐fatal overdose were also recorded. Risky alcohol use was defined using the Alcohol Use Disorder Identification Test (AUDIT‐C) [30], a score of 4 or more for men and 3 or more for women.

Severity of opioid dependence was measured using questions adapted from the World Health Organization Composite International Diagnostic Interview substance use module [31], mapped to DSM‐5 opioid use disorder criteria and analogous ICD dependence‐syndrome constructs. Eleven symptom items assessed craving, tolerance, withdrawal, impaired control, time spent using, reduction in important activities and continued use despite harms and symptom counts over the past 12 months were used to classify mild (2–3 criteria), moderate (4–5) and severe (≥ 6) opioid use disorder [32]. Opioid use disorder severity was utilised rather than opioid dependence, due to ease of categorisation.

Moderate to severe depression was defined as a score of 10 or more on the Patient Health Questionnaire (PHQ‐9) [33]. High risk of suicidal behaviour was defined as a score of 21 or more on the Suicidal Ideation Attributes Scale (SIDAS) [34]. Moderate to severe anxiety was defined as a score of 10 or more on the Generalised Anxiety Disorder Scale (GAD‐7) [35]. Post‐traumatic stress disorder (PTSD) was classified as a score of 4 or more on the Primary Care PTSD Screen for DSM‐5 (PC‐PTSD‐5) [36]. Questions used to define ACEs were adapted from the Adverse Childhood Experiences Questionnaire (ACE‐Q) [37].

#### 2.3.2 Aim 2: OAT Characteristics

Participants receiving OAT at the time of the survey were asked about their OAT medication type, dose, dosing point, frequency of visits in the past month (daily/close to daily; several times per week; weekly to fortnightly; monthly or less), distance (kilometres, km) and travel time (minutes) to pick‐up medication and the cost per visit ($AUD).

#### 2.3.3 Aim 3: Engagement With Harm Reduction and Support Services

Engagement with harm reduction and support services was assessed using a series of items. Harm reduction indicators included lifetime use of NSP and lifetime use of the medically supervised injecting centre, as well as use of NSP and Medically Supervised Injecting Centre in the past 12 months. Receipt of naloxone kit(s) in the past 12 months and currently carrying naloxone were also recorded for all participants.

We additionally measured current engagement with other support services, including group therapy for substance dependence, group therapy for mental health, individual counselling for substance use, individual counselling for mental health, parenting support, financial counselling, housing support, and education or employment support.

### 2.4 Statistical Analysis

Quantitative data were analysed using R Version 4.2.3 [38] and STATA software version 18 [39]. Categorical variables were expressed as percentages. Continuous variables were presented as means (standard deviation) where normally distributed and medians (inter‐quartile range), where non‐normally distributed. Categorical variables were compared using the *χ* ^2^ test, except for variables with a cell count < 5, in which case Fisher's exact test was used. Continuous variables were compared using an independent samples *t*‐test.

Participants were grouped by OAT status: ‘currently receiving OAT’, ‘previously received OAT’, which included those who were not currently receiving OAT but had received OAT at least once in their lifetime and ‘never received OAT’. Results from Aim 1 analyses are presented in Tables 1, 2, 3. Group comparisons follow the tests described above. To account for multiple comparisons, Benjamini and Hochberg false discovery rate correction with *Q* = 0.05 was applied separately within each of Tables 1, 2, 3 [40]. Variables that remained statistically significant after false discovery rate correction are marked within the tables.

**TABLE 1:** Demographic characteristics by opioid agonist treatment status among a sample of people with opioid dependence in New South Wales, Australia (*N* = 403).

Variable	Overall, *N* = 403		Participants currently receiving OAT (A), *N* = 312		Participants previously receiving OAT (B), *N* = 53		Participants never received OAT (C), *N* = 38		*p* (A vs. B)^a^	*p* (A vs. C)^a^
	*n*	% (95% CI)	*n*	% (95% CI)	*n*	% (95% CI)	*n*	% (95% CI)		
Mean age (SD)	44 (10)	—	44 (10)	—	44 (10)	—	42 (8)	—	0.709	0.256
Gender									0.438	0.213
Male	261	65% (60%–69%)	193	62% (56%–67%)	38	72% (58%–82%)	30	79% (62%–89%)		
Female	136	34% (29%–39%)	114	37% (31%–42%)	14	26% (16%–40%)	8	21% (11%–38%)		
Non‐binary	—	—	—	—	—	—	—	—		
Other	—	—	—	—	—	—	—	—		
Sexual orientation^c^									0.194	0.611
Straight/heterosexual	328	83% (79%–86%)	252	82% (77%–86%)	45	85% (72%–92%)	31	86% (70%–94%)		
Gay/lesbian	19	5% (3%–7%)	16	5% (3%–8%)	—^b^	—^b^	—^b^	—^b^		
Bisexual	41	10% (8%–14%)	35	11% (8%–15%)	—^b^	—^b^	—^b^	—^b^		
Other	9	2% (1%–4%)	5	2% (1%–4%)	—^b^	—^b^	—^b^	—^b^		
Education < Year 10	130	32% (28 − 37%)	99	32% (27 − 37%)	16	30% (19%–44%)	15	39% (25%–56%)	0.874	0.363
Unemployed	342	85% (81%–88%)	267	86% (81%–89%)	44	83% (70%–91%)	31	82% (65%–91%)	0.535	0.469
Region of residence									**0.031**	**0.001** ^d^
Sydney	300	75% (70%–79%)	219	71% (65%–75%)	45	85% (72%–92%)	36	95% (80%–99%)		
Other NSW region	101	25% (21%–30%)	91	29% (25%–35%)	—^b^	—^b^	—^b^	—^b^		
Homeless/no fixed residence	59	15% (12%–19%)	32	10% (7%–14%)	15	28% (18%–42%)	12	32% (18%–49%)	**0.001** ^d^	**0.001** ^d^
Pension/disability benefit	180	45% (40%–50%)	146	47% (41%–52%)	22	42% (29%–56%)	12	32% (18%–49%)	0.552	0.085
Incarcerated ever^c^	256	67% (62%–72%)	198	67% (52%–72%)	39	75% (61%–85%)	19	58% (40%–74%)	0.332	0.332
Incarcerated past 12 months^c^	74	19% (16%–24%)	53	18% (14%–23%)	15	29% (18%–43%)	6	18% (8 − 36%)	0.087	1.000

*Note:* Bold value indicates *p* < 0.05.

Abbreviations: CI, confidence interval; NSW, New South Wales; OAT, opioid agonist treatment; SD, standard deviation.

^a^ *t*‐test, Pearson's *χ* ^2^ test, Fisher's exact test.

^b^ Data suppressed to protect participant confidentiality.

^c^ Missing data: sexual orientation (*n* = 6, 1.5%), Incarcerated ever/past 12 months (*n* = 23, 5.7%) and other variables < 1%.

^d^ Significance after Benjamini–Hochberg FDR correction (*q* < 0.05) applied.

**TABLE 2:** Substance use and related experiences among a sample of people with opioid dependence in New South Wales, Australia by opioid agonist treatment (OAT) status (*N* = 403).

	*N* = 403		Participants currently receiving OAT (A), *N* = 312		Participants previously receiving OAT (B), *N* = 53		Participants never received OAT (C), *N* = 38			
	*n*	% (95% CI)	*n*	% (95% CI)	*n*	% (95% CI)	*n*	% (95% CI)	*p* (A vs. B)^a^	*p* (A vs. C)^a^
Injecting drug use										
Ever	380	95% (92%–96%)	299	96% (93%–98%)	52	98% (87%–100%)	29	76% (60%–88%)	0.702	< **0.001** ^f^
Past 12 months	324	80% (76%–84%)	249	80% (75%–84%)	50	94% (83%–98%)	25	66% (49%–80%)	**0.011** ^f^	**0.048**
Past month	258	64% (59%–69%)	186	60% (54%–65%)	47	89% (77%–95%)	25	66% (49%–80%)	**< 0.001** ^f^	0.463
Daily in past month	158	39% (35%–44%)	102	33% (28%–38%)	37	70% (56%–81%)	19	50% (34%–66%)	**< 0.001** ^f^	**0.034**
DSM‐5 opioid use disorder^d^									**0.031**	0.116
None										
Mild	27	7% (5%–10%)	26	8% (6%–12%)	—^c^	—^c^	—^c^	—^c^		
Moderate	31	8% (5%–11%)	25	8% (5%–12%)	—^c^	—^c^	—^c^	—^c^		
Severe	276	68% (64%–73%)	201	64% (59%–70%)	45	85% (72%–93%)	30	79% (62%–90%)		
Past month extra‐medical use										
Heroin	247	61% (56%–66%)	168	54% (48%–59%)	49	92% (81%–97%)	30	79% (62%–89%)	< **0.001** ^f^	**0.003** ^f^
Methadone	76	19% (15%–23%)	53	17% (13%–22%)	18	34% (22%–48%)	5	13% (5%–29%)	**0.004** ^f^	0.549
Buprenorphine (no naloxone)	22	5% (4%–8%)	7	2% (1%–5%)	6	11% (5%–24%)	9	24% (12%–40%)	**0.001** ^f^	**< 0.001** ^f^
Buprenorphine with naloxone	34	8% (6%–12%)	13	4% (2%–7%)	11	21% (12%–34%)	10	26% (14%–43%)	**< 0.001** ^f^	**< 0.001** ^f^
Oxycodone	38	9% (7%–13%)	15	5% (3%–8%)	10	19% (10%–32%)	13	34% (20%–51%)	**< 0.001** ^f^	**< 0.001** ^f^
Morphine	22	5% (4%–8%)	13	4% (2%–7%)	—^c^	—^c^	5	13% (5%–29%)	0.287	**0.018** ^f^
Codeine	39	10% (7%–13%)	19	6% (4%–9%)	8	15% (8%–28%)	12	32% (18%–49%)	**0.021** ^f^	**< 0.001** ^f^
Tramadol	10	2% (1%–5%)	5	2% (1%–4%)	—^c^	—^c^	—^c^	—^c^	0.270	**0.045**
Fentanyl	17	4% (3%–7%)	13	4% (2%–7%)	—^c^	—^c^	—^c^	—^c^	0.702	0.398
Benzodiazepines	92	23% (19%–27%)	67	21% (17%–26%)	17	32% (21%–46%)	8	21% (11%–38%)	0.090	0.952
Cocaine	51	13% (10%–16%)	38	12% (9%–16%)	8	15% (8%–28%)	5	13% (5%–29%)	0.554	0.862
Meth/amphetamine	179	44% (40%–49%)	122	39% (34%–45%)	34	64% (50%–76%)	23	61% (44%–75%)	**0.001** ^f^	**0.011** ^f^
Alcohol use disorder—AUDIT‐C^b^	154	38% (34%–43%)	117	38% (32%–43%)	20	38% (25%–52%)	17	45% (29%–61%)	0.974	0.386
Overdose										
Ever^e^	237	63% (58%–68%)	188	64% (59%–70%)	34	65% (51%–77%)	15	48% (31%–66%)	0.889	0.080
Past 6 months^e^	40	11% (8%–14%)	25	9% (6%–12%)	11	21% (12%–35%)	—^c^	—^c^	**0.006** ^f^	0.502

*Note:* Bold value indicates *p* < 0.05.

Abbreviations: CI, confidence interval; OAT, opioid agonist treatment.

^a^ Pearson's *χ* ^2^ test, Fisher's exact test.

^b^ Score of 4 or more for men, 3 or more for women on the Alcohol Use Disorder Identification Test (AUDIT‐C).

^c^ Data suppressed to protect participant confidentiality.

^d^ Classified using the Diagnostic and Statistical Manual of Mental Disorders, Fifth Edition (DSM‐5) criteria for Diagnosis of Opioid Use Disorder.

^e^ Missing data: overdose ever (*n* = 28, 6.9%), overdose Past 6 months (*n* = 27, 6.7%).

^f^ Significance after Benjamini–Hochberg FDR correction (*q* < 0.05) applied.

**TABLE 3:** Mental health and related experiences among a sample of people with opioid dependence in New South Wales, Australia, by opioid agonist treatment (OAT) status (*N* = 403).

Variable	Overall, *N* = 403		Participants currently receiving OAT (A), *N* = 312		Participants previously receiving OAT (B), *N* = 53		Participants never received OAT (C), *N* = 38			
	*n*	% (95% CI)	*n*	% (95% CI)	*n*	% (95% CI)	*n*	% (95% CI)	*p* (A vs. B)^a^	*p* (A vs. C)^a^
Post‐traumatic stress disorder (PC‐PTSD‐5)^b^, ^k^	141	49% (43%–55%)	114	40% (34%–45%)	17	36% (23%–51%)	10	31% (17%–50%)	0.531	0.669
Depression, moderate%–severe (PHQ‐9)^c^, ^k^	204	54% (49%–59%)	160	54% (49%–60%)	25	51% (37%–65%)	19	58% (40%–74%)	0.658	0.730
High risk of suicidal behaviour^d^, ^k^	30	8% (6%–11%)	22	7% (5%–11%)	5	10% (4%–23%)	—^e^	—^e^	0.508	0.733
Anxiety, moderate–severe (GAD‐7)^f^, ^k^	139	38% (33%–43%)	110	38% (33%–44%)	19	40% (26%–54%)	10	32% (18%–51%)	0.869	0.508
Adverse childhood experiences (ACE)^g^										
Any ACE^k^	299	91% (88%–94%)	234	92% (88%–95%)	43	93% (81%–98%)	20	77% (56%–90%)	1.000	**0.008**
Abuse^h^, ^k^	235	72% (67%–77%)	189	75% (69%–80%)	28	62% (47%–76%)	17	65% (44%–82%)	0.083	0.303
Neglect^i^, ^k^	195	61% (55%–66%)	159	63% (57%–69%)	23	51% (36%–66%)	13	52% (32%–72%)	0.128	0.275
Household dysfunction^j^, ^k^	268	83% (78%–87%)	212	84% (79%–88%)	38	86% (72%–94%)	17	65% (44%–82%)	0.706	**0.017**
Psychiatrist visit in past 12 months^k^	104	27% (23%–31%)	81	27% (23%–33%)	13	25% (15%–40%)	6	19% (9%–38%)	0.771	0.332
Psychologist visit in past 12 months^k^	128	33% (28%–38%)	102	35% (29%–40%)	15	29% (18%–44%)	7	23% (11%–42%)	0.462	0.174

*Note:* Bold value indicates *p* < 0.05. Benjamini–Hochberg FDR correction was applied to the set of pairwise comparison *p* values within each comparison column; no associations remained significant after correction at *q* < 0.05.

Abbreviation: CI, confidence interval.

^a^ Pearson's *χ* ^2^ test, Fisher's exact test.

^b^ Score of 4 or more on the Primary Care PTSD Screen for DSM‐5 (PC‐PTSD‐5).

^c^ Score of 10 or more on the Patient Health Questionnaire‐9 (PHQ‐9).

^d^ Score of 21 or more on the Suicidal Ideation Attributes Scale (SIDAS).

^e^ Data suppressed to protect participant confidentiality.

^f^ Score of 10 or more on the General Anxiety Disorder‐7 (GAD‐7).

^g^ Questions used to define ACEs were adapted from the Adverse Childhood Experiences Questionnaire (ACE‐Q).

^h^ Defined by Questions 1–3 from the ACE‐Q.

^i^ Defined by Questions 4–5 from the ACE‐Q.

^j^ Defined by Questions 6–10 from the ACE‐Q.

^k^ Missing data: post‐traumatic stress disorder (*n* = 116, 28.8%), depression (*n* = 27, 6.7%), high risk of suicidal behaviour (*n* = 25, 6.2%), anxiety (*n* = 37, 9.2%), any ACE (*n* = 76, 18.9%), ACE Abuse (*n* = 78, 19.4%), ACE neglect (*n* = 81, 20.1%), ACE household dysfunction (*n* = 80, 19.9%), psychiatrist/psychologist (*n* = 13, 3.2%).

Aim 2 is restricted to participants who were currently receiving OAT and is presented descriptively in Table 4. This table summarises characteristics of the current OAT episode, including medication type, dose, dosing location, visit frequency, travel distance and time, out of pocket fees and concurrent substance use, stratified by methadone, sublingual buprenorphine or buprenorphine naloxone and long‐acting injectable buprenorphine (LAIB). No formal hypothesis tests were conducted for Aim 2.

**TABLE 4:** Characteristics of current opioid agonist treatment (OAT) episode among participants currently receiving OAT, by medication type (*N* = 303^a^).

	Methadone		Sublingual buprenorphine/buprenorphine‐naloxone		Long‐acting injectable buprenorphine	
	*n*	% (95% CI)	*n*	% (95% CI)	*n*	% (95% CI)
Number of participants (%)	186	60% (55%–66%)	34	11% (8%–15%)	83	27% (23%–33%)
Median dose (daily or weekly/monthly) (mg) [Median] (IQR)	184	65 (22%–103)	33	16 (8%–32)	80	96 (64%–128)
Location of most recent dose						
Public clinic/hospital	60	32% (26%–40%)	17	50% (33%–67%)	43	52% (41%–63%)
Pharmacy	74	40% (33%–48%)	—^b^	—^b^	—^b^	—^b^
Private clinic	28	15% (11%–21%)	—^b^	—^b^	—^b^	—^b^
Other	22	12% (8%–18%)	6	18% (8%–35%)	27	33% (23%–44%)
Frequency of visits to receive dose in past 28 days						
Daily or close to daily	102	55% (48%–63%)	—^b^	—^b^	—^b^	—^b^
Several times per week	53	28% (23%–36%)	—^b^	—^b^	—^b^	—^b^
Weekly to fortnightly	27	15% (10%–21%)	—^b^	—^b^	—^b^	—^b^
Monthly or less	—^b^	—^b^	—^b^	—^b^	62	75% (68%–86%)
Distance to pickup location (km) [Median] (IQR)	177	3 (1–10)	32	5 (2–13)	73	3 (1–5)
Travel time to pickup location (minutes) [Median time]	185	10 (5–25)	34	15 (7–30)	82	10 (2–20)
Pay out‐of‐pocket fees? (yes)	93	50% (43%–57%)	16	47% (30%–64%)	9	11% (6%–20%)
Cost of treatment in AUD [Median cost] (IQR)	93	$8 ($7–$30) per visit	16	$8 ($7–$23) per visit	8	$9 ($7–$54) per visit
Past month substance use						
Heroin	106	57% (50%–64%)	17	50% (32%–68%)	39	47% (36%–58%)
Methadone	46	25% (19%–32%)	—^b^	—^b^	—^b^	—^b^
Subutex	—^b^	—^b^	—^b^	—^b^	—^b^	—^b^
Suboxone	—^b^	—^b^	9	26% (14%–45%)	—^b^	—^b^
Oxycodone	8	4% (2%–8%)	—^b^	—^b^	—^b^	—^b^
Morphine	8	4% (2%–8%)	—^b^	—^b^	—^b^	—^b^
Codeine	—^b^	—^b^	—^b^	—^b^	8	10% (5 — 18%)
Tramadol	—^b^	—^b^	—^b^	—^b^	—^b^	—^b^
Fentanyl	—^b^	—^b^	—^b^	—^b^	6	7% (3 − 15%)
Benzodiazepines	40	22% (16%–28%)	5	15% (6%–32%)	12	14% (8%–24%)
Cocaine	18	10% (6%–15%)	6	18% (7%–35%)	12	14% (8%–24%)
Meth/amphetamine	62	33% (27%–41%)	16	47% (30%–64%)	40	48% (37%–59%)

Abbreviations: AUD, alcohol use disorder; CI, confidence interval; IQR, interquartile range.

^a^ *n* = 9 (2.9%) missing data for variable current type of OAT received.

^b^ Data suppressed to protect participant confidentiality.

Aim 3 examines engagement with harm reduction and other support services and is presented in Table 5. Ever and past 12‐month use of NSPs and the medically supervised injecting centre were analysed among participants for whom each outcome was relevant (ever injecting for ever use outcomes and injecting in the past 12 months for past 12‐month outcomes). Other support service variables, including receipt of naloxone kit or kits in the past 12 months, currently carrying naloxone and current use of group or individual therapies and social support services, were analysed in the full analytic sample. For these outcomes we estimated odds ratios and 95% confidence intervals comparing ‘participants currently receiving OAT’ with ‘participants previously receiving OAT’ and with ‘participants who had never received OAT’, using unadjusted and age and sex adjusted logistic regression models, with age entered as a continuous variable.

**TABLE 5:** Engagement with harm reduction and support services by OAT status among people with opioid dependence (*N* = 403).

	*N* (403)	Currently receiving OAT (A), *N* = 312	Previously received OAT (B), *N* = 53	Never Received OAT (C)*, N* = 38	Odds ratio (95% CI), (A vs. B)	Adjusted odds ratio (95% CI), (A vs. B)^a^	Odds ratio (95% CI), (A vs. C)	Adjusted odds ratio (95% CI), (A vs. C)^a^
Needle and syringe program								
Ever used	307 (76%)	245 (79%)	44 (83%)	18 (47%)	0.86 (0.34, 2.14)	0.86 (0.34, 2.18)	2.44 (0.96, 6.23)	2.57 (0.97, 6.80)
Past 12‐month use	244 (61%)	192 (62%)	36 (68%)	16 (42%)	1.88 (0.69, 5.10)	1.51 (0.52, 4.37)	0.71 (0.09, 5.65)	0.67 (0.08, 5.38)
Safe injecting room								
Ever used	204 (51%)	155 (50%)	34 (64%)	15 (39%)	0.58 (0.31, 1.11)	0.59 (0.31, 1.12)	0.83 (0.36, 1.90)	0.84 (0.36, 1.96)
Past 12‐month use	98 (24%)	69 (22%)	18 (34%)	11 (29%)	0.77 (0.36, 1.64)	0.69 (0.31, 1.53)	**0.25 (0.07, 0.94)**	**0.25 (0.07, 0.97)**
Received naloxone kit/s in past 12‐months	227 (56%)	183 (59%)	32 (60%)	12 (32%)	0.99 (0.53, 1.83)	0.92 (0.49, 1.73)	**2.77 (1.30, 5.89)**	**2.58 (1.19, 5.57)**
Carries naloxone currently	78 (19%)	67 (21%)	—^c^	—^c^	1.29 (0.58, 2.90)	1.25 (0.55, 2.83)	Not reported^b^	Not reported^b^
Other support services								
Group therapy for substance dependence	86 (21%)	69 (22%)	—^c^	—^c^	0.72 (0.37, 1.38)	0.71 (0.36, 1.38)	**5.11 (1.20, 21.76)**	**5.97 (1.38, 25.81)**
Group therapy for mental health	56 (14%)	43 (14%)	—^c^	—^c^	0.69 (0.32, 1.47)	0.65 (0.30, 1.42)	1.86 (0.55, 6.33)	2.00 (0.57, 6.97)
Individual counselling for substance use	110 (27%)	88 (28%)	16 (30%)	6 (16%)	0.91 (0.48, 1.72)	0.86 (0.45, 1.64)	2.10 (0.85, 5.19)	2.04 (0.82, 5.09)
Individual counselling for mental health	122 (30%)	97 (31%)	17 (32%)	8 (21%)	0.96 (0.51, 1.78)	0.88 (0.46, 1.67)	1.69 (0.75, 3.83)	1.60 (0.70, 3.67)
Parenting support	30 (7%)	27 (9%)	—^c^	—^c^	Not reported^a^	Not reported^a^	1.11 (0.32, 3.83)	0.94 (0.26, 3.39)
Financial counselling	26 (6%)	20 (6%)	—^c^	—^c^	1.75 (0.40, 7.70)	1.55 (0.35, 6.91)	0.58 (0.19, 1.80)	0.51 (0.16, 1.61)
Housing support	136 (34%)	107 (34%)	11 (21%)	18 (47%)	2.0 (0.99, 4.03)	1.88 (0.92, 3.82)	0.58 (0.30, 1.14)	0.51 (0.25, 1.02)
Education/employment support	71 (18%)	56 (18%)	7 (13%)	8 (21%)	1.44 (0.62, 3.4)	1.36 (0.58, 3.20)	0.82 (0.36, 1.88)	0.77 (0.33, 1.80)

*Note:* Bold value indicates *p* < 0.05.

Abbreviations: CI, confidence interval; OAT, opioid agonist treatment.

^a^ Age and sex adjusted.

^b^ Odds ratios were unable to be generated due to small or zero value.

^c^ Data suppressed to protect participant confidentiality.

Table cell counts less than five have been suppressed to protect participant confidentiality. Missing data are detailed within each table, and a complete case analysis approach was used. Findings are reported in accordance with the STROBE checklist (Appendix B).

### 2.5 Ethical Approval

The study was approved by the St Vincent's Hospital Sydney Human Research Ethics Committee (2023/ETH01404) and the Corrective Services NSW Ethics Committee and conducted in accordance with the National Statement on Ethical Conduct in Human Research [41]. All participants were provided with written information about the study and gave written informed consent before enrolment. We obtained access approvals from local health districts: Central Coast, Hunter New England, Illawarra Shoalhaven, Nepean Blue Mountains, Mid North Coast, Northern NSW, South East Sydney, Sydney, Western NSW, Northern Sydney and the Community Mental Health, Drug and Alcohol Research Network Research Ethics Consultation Committee.

## 3 Results

### 3.1 Sample Characteristics

Of the 540 people screened, 403 participants were eligible and reported their OAT status. The mean age was 44 years (SD = 10; Table 1). The cohort was predominantly male (65%), identified as straight/heterosexual (83%) and had completed year 10 education (68%). Current unemployment was common (85%) and 15% were homeless or had no fixed residence.

Past month extra‐medical opioid use was highest for heroin (61%), followed by methadone (19%), and codeine (10%) (Table 2). Lifetime overdose was reported by 63% of the sample. Over half (54%) of participants reported current moderate–severe depression, and almost half reported current PTSD (49%) and moderate–severe anxiety (38%). Participants currently receiving OAT comprised 77% (*n* = 312) of the sample, with 13% (*n* = 53) having previously received OAT and 9% (*n* = 38) having never received OAT.

### 3.2 Socio‐Demographic, Substance Use and Mental Health Characteristics According to Current OAT Status

There were no significant differences in most of the sociodemographic variables examined according to OAT history, with the exception that those who had never or were not currently receiving OAT were more likely to be experiencing homelessness or have no current fixed address and were more likely to live in Sydney than in another NSW region, although the regional difference between current and previous‐OAT groups was not significant after adjusting for multiple comparisons (Table 1).

Daily injecting drug use in the past month was lower among participants currently receiving OAT (33%) compared to those formerly (70%) or never (50%) in OAT, although the difference between current and never‐OAT groups was not significant after adjusting for multiple comparisons (Table 2). Similar trends were observed for past‐month heroin use (previously: 92%; never: 79%; current: 54%). Additionally, past month extra‐medical use of sublingual buprenorphine was more common among those never receiving OAT (buprenorphine without naloxone: 24%; buprenorphine with naloxone: 26%) compared to the current OAT group (buprenorphine without naloxone: 2%; buprenorphine with naloxone: 4%). Past‐month methamphetamine use was significantly higher in the never‐OAT group (61%) and former‐OAT group (64%) compared to those currently in OAT (39%). Severe opioid use disorder was also more common among those previously in OAT (85%) compared to those currently in OAT (64%). Past‐6‐month overdose was lower among those receiving OAT (9%) compared to those previously in OAT (21%; *p* = 0.006). There was no significant association with current OAT status in levels of mental health disorders (Table 3).

### 3.3 Characteristics of Current OAT

Among those currently receiving OAT, methadone was the most common form of OAT (60%; Table 4), followed by LAIB at 27% and sublingual buprenorphine at 11%. The location of participants' last dose was most commonly public clinics or hospitals for those receiving LAIB (52%) and sublingual buprenorphine (50%) and pharmacies for those receiving methadone (40%). In the month before the survey, individuals receiving methadone or oral buprenorphine were most likely to be receiving daily supervised doses (55% and *suppressed*, respectively), whereas those receiving LAIB largely received their doses monthly (75%). Among participants currently or previously receiving OAT, lifetime history of residential rehabilitation and outpatient counselling were 64% and 54%, respectively (Appendix C).

### 3.4 Other Substance Use Treatment, Harm Reduction and Mental Health Treatment Use

Table 5 shows the use of harm reduction and other support services. In the past year, 61% had used an NSP and 56% had received naloxone, of which one in five (19%) reported currently carrying it with them. Safe injecting rooms were less frequently attended in the past year (24%). In the past year, participants reported using various support services, including housing support (34%), mental health counselling (30%), individual (27%) and group (21%) therapy for substance dependence, and education or employment support (18%).

Participants currently receiving OAT reported higher odds of receiving naloxone kits in the past 12 months and engaging with group therapy for substance dependence compared to participants who had previously or never received OAT. In comparison, participants currently receiving OAT had lower odds of past‐year safe injecting room use than participants who reported previously or never receiving OAT.

## 4 Discussion

Our study provides a detailed examination of the socio‐demographic, mental health, substance use and service use profiles of people with opioid dependence in NSW after reductions in dosing fees and the wider availability of LAIB. By contrasting current, former and never engagement with OAT, documenting contemporary prescribing patterns for methadone and buprenorphine and mapping participation in harm reduction and support programs, our findings provide critical insights into how recent NSW reforms are reflected in delivery of opioid dependence treatment and identify gaps in both treatment and harm reduction service access.

Our findings indicate the high rate of issues that are often not recorded in routinely collected OAT data [42], such as unemployment and unstable housing. Effective service provision requires integrating into care models strategies for addressing the social and environmental factors affecting this population. Initiatives such as the NSW Opioid Treatment Program Transition to Section 100 Highly Specialised Drugs Program [43] aim to reduce the burden of costs to clients receiving OAT through covering costs of pharmacy OAT provision, yet much more is needed [43]. A major barrier to resolving unstable housing or homelessness is a lack of access to government supported accommodation, which has long‐waiting lists for places that tend to be in increasingly less central areas [44]. Structural factors that exist as barriers to obtaining employment—and consequently, stable and supportive housing in this population, include lower levels of education, as well as limited access and exposure to training [45]. Specific strategies to address these structural barriers could include coordinated care through community‐based services to support expanded access to education, training, temporary housing, and work experience [46]. Future research linking interview data with treatment and social service records will support more detailed insights on opportunities for providing and evaluating interventions to support long‐term health and social outcomes in this population.

Among the survey participants receiving OAT, approximately one in four were receiving LAIB, reflecting the uptake of this newer treatment option. The distribution of OAT medication type in our treatment sample broadly aligns with the National Opioid Pharmacotherapy Statistics Annual Data (NOPSAD), which provides a snapshot of all people receiving OAT on a single day in NSW. While methadone use was more common in our sample (60% vs. 48% in NOPSAD), there was an equal uptake of LAIB (27% in both our survey and NOPSAD) [47]. Many participants on methadone reported daily or near‐daily attendance for dosing, which likely reflects the reinstated supervised‐dosing constraints—now capped at two to four take‐away doses per week depending on patient risk, following the temporary COVID‐19 flexibilities [21, 48, 49, 50]. Additionally, almost two‐thirds of individuals receiving OAT had been to residential rehabilitation, highlighting the potential role of these services in linking people with opioid dependence to longer‐term treatment, such as OAT and services for other conditions in NSW.

Our findings revealed that participants not receiving OAT experienced higher levels of homelessness and were more likely to report recent heroin use and daily injecting. This is consistent with evidence that entry and retention in OAT is associated with reductions in extra‐medical opioid use, injecting drug use and improved social well‐being [1]. Furthermore, differences were evident in polysubstance use by treatment engagement; those currently receiving OAT reported lower rates of these high‐risk behaviours compared to those who had stopped or never received OAT. To address these disparities, improving overdose prevention and harm reduction services outside of treatment settings is crucial [51]. This includes expanding access to naloxone to prevent overdose deaths, increasing the availability of supervised injecting facilities to provide safer environments and offering broader community support programs such as access to peer harm reduction information, housing assistance and mental health services. These interventions can significantly reduce harm and improve the overall health and well‐being of those living with opioid dependence [52, 53, 54].

The burden of mental disorders and trauma in this cohort was substantial. Around half of participants reported current moderate to severe depressive symptoms, one‐third reported moderate to severe anxiety, and around half screened positive for probable PTSD, with high levels of suicidal ideation and exposure to ACE also reported. These findings are consistent with previous work among people with opioid dependence in NSW, which demonstrated that childhood trauma and mental disorders are strongly associated with slower initiation to OAT, lower rates of treatment engagement and higher rates of contact with the criminal justice system [55]. A substantial proportion of participants also reported current contact with psychiatrists, psychologists or other counsellors, indicating some engagement with mental health care, yet the high symptom burden suggests that unmet need is likely to remain. We did not observe consistent differences in the prevalence of these mental health disorders by OAT status, suggesting that mental health needs are pervasive among people with opioid dependence, regardless of whether they are currently in treatment. This underscores the importance of integrating evidence‐based mental health assessment and care or robust referral pathways to psychiatric and psychological care for people with opioid dependence within facilities that administer OAT and harm reduction services. As uptake of LAIB increases and the frequency of supervised dosing visits declines, models of care will need to ensure that reduced dosing contact does not inadvertently diminish opportunities to identify and address mental health and trauma‐related needs for people with opioid dependence.

Although this study provides valuable insights into individuals with opioid dependence and access to OAT in NSW, several limitations should be acknowledged. The non‐probability sampling approach, which relied on word of mouth, community organisations, harm reduction services, OAT dosing sites and engagement with local health district services, likely overrepresents individuals who have frequent contact with treatment and harm reduction services, such as those receiving methadone with regular supervised dosing. In contrast, the sample may underrepresent people from rural areas and those with less frequent service engagement. Individuals engaged with treatment or harm reduction services often report elevated rates of psychosocial concerns, including mental and substance use disorders [2, 56]; therefore, rates observed in this study may be higher than those in the broader population of people with opioid dependence. Nevertheless, the relatively large sample size and diverse recruitment strategies across 12 local health districts, supplemented by outreach through community pharmacies, private OAT clinics, rural pharmacies, mail‐order services and a 5‐month recruitment window, likely mitigate concerns regarding representativeness.

Furthermore, results from this study are not necessarily representative of all people with opioid dependence in NSW, especially as it relates to the gender composition of the sample. The well‐documented gender differences in substance use patterns, experiences of violence and trauma, treatment access and co‐occurring psychiatric disorders mean that addressing these gaps is critical to ensure that the needs of women and gender‐diverse people with opioid dependence are adequately captured. Additionally, several subgroup comparisons relied on small cell counts that produced wide confidence intervals and limited statistical power. Reliance on self‐report may also introduce recall bias and missing data for sensitive experiences such as childhood adversity, mental disorders and suicidal behaviour, because stigma or distress can lead to under‐reporting [57, 58].

This study set out to describe the current profile of opioid dependence in NSW, to document how OAT is delivered, and to map the use of harm‐reduction and other supports. The findings show clear differences by treatment status. People who were not receiving OAT reported greater housing instability, more frequent injecting and heroin use, and were less likely to obtain naloxone. Although the roll‐out of LAIB and lower dispensing fees for community OAT may have eased some barriers, many individuals are not receiving treatment but are in contact with existing harm‐reduction services. Extending low‐threshold pathways into OAT, particularly, in community and rural settings, and linking treatment more routinely with housing, mental‐health and employment assistance are necessary steps if recent policy reforms are to translate to improved health service engagement and health outcomes among people with opioid dependence in NSW.

## Author Contributions

Each author certifies that their contribution to this work meets the standards of the International Committee of Medical Journal Editors.

## Funding

This study was supported by an Australian National Health and Medical Research Council (NHMRC) Program grant (1150078), an National Health & Medical Research Council (1176131, 2016825, 2034002, and 1135991) to L.D.; National Institute on Drug Abuse (1R01DA059822‐01A1) and the National Drug and Alcohol Research Centre, UNSW Sydney and Department of Health and Aged Care, Australian Government. The National Drug and Alcohol Research Centre is funded by the Australian Government Department of Health. The views expressed in this publication do not necessarily represent the position of the Australian Government.

## Conflicts of Interest

Past 3 years, J.G. is a consultant or advisor and has received research grants from Abbvie, Abbott, bioLytical, Cepheid, Gilead Sciences, Hologic and Roche. The other authors declare no conflicts of interest.

## Data Availability

Research data are not shared.

## Appendix A See Table A1

**TABLE A1:** Summary of survey question domains and questions from the NSW Opioid Dependence Survey.

Domain	Key questions/details
Demographics	Age, postcode, education, gender, sexual orientation, intersex variation, sex at birth, employment, income sources, medicare card, healthcare card
Housing and environment	Current residence, homelessness experience, timing of last homelessness
Substance use history	First illicit substance, age of first use, method of first use, injection history, recent injection, injection equipment source, illicit opioid use, substance use details
Alcohol use (AUDIT‐C)	Frequency of drinking, typical quantity, frequency of binge drinking
Opioid dependence (DSM‐5)	Criteria for opioid use disorder (e.g., tolerance, withdrawal, unplanned use, continued use despite problems)
Opioid agonist treatment	Medication history, current treatment, type and dose, treatment duration, satisfaction, dispensation location, visit frequency, missed doses, travel for treatment, costs, concurrent drug use
Treatment preferences	Discussion and availability of treatment options, preferred medications, interest in alternative treatments (e.g., SIOT, Hydromorphone), access barriers
Quality of life (EQ‐5D‐5L)	Mobility, self‐care, usual activities, pain/discomfort, anxiety/depression, overall health rating
Mental health	Medication history, psychiatrist/psychologist visits, Patient Health Questionnaire (PHQ‐9), Suicidal Ideation Attributes Scale (SIDAS), Generalised Anxiety Disorder Scale (GAD‐7), Primary Care PTSD Screen (PC‐PTSD‐5)
Adverse childhood experiences	Experiences of abuse, neglect, household dysfunction before age 18
Harm reduction services	Needle syringe program use, safe injecting room use, other support services received or preferred (e.g., therapy, financial counselling, housing support)
Overdose history	Overdose experiences, overdose responses, naloxone usage and possession, contexts and locations, actions taken during overdose, outcomes
Incarceration and arrest	Incarceration history, age at first incarceration, duration, reason for recent incarceration, arrests for opioid use/possession, treatment during incarceration, drug use in incarceration
Physical health	Skin and soft tissue infections (treatment and hospitalisation), hepatitis C testing and treatment, HIV testing and treatment

*Note:* Does not include all survey questions.

Abbreviations: AUDIT‐C, Alcohol Use Disorder Identification Test; DSM‐5, Diagnostic and Statistical Manual of Mental Disorders, Fifth Edition; NSW, New South Wales; SIOT, supervised injectable opioid oreatment.

## Appendix B See Table B1

**TABLE B1:** STROBE Statement for cross‐sectional studies.

	Item no	Recommendation	Page
Title and abstract	1	(a) Indicate the study's design with a commonly used term in the title or the abstract	1
		(b) Provide in the abstract an informative and balanced summary of what was done and what was found	2
Introduction			
Background/rationale	2	Explain the scientific background and rationale for the investigation being reported	5
Objectives	3	State specific objectives, including any prespecified hypotheses	6
Methods			
Study design	4	Present key elements of study design early in the paper	7–10
Setting	5	Describe the setting, locations, and relevant dates, including periods of recruitment, exposure, follow‐up and data collection	7–10
Participants	6	(a) Give the eligibility criteria, and the sources and methods of selection of participants	7–10
Variables	7	Clearly define all outcomes, exposures, predictors, potential confounders and effect modifiers. Give diagnostic criteria, if applicable	7–10
Data sources/measurement	8	For each variable of interest, give sources of data and details of methods of assessment (measurement). Describe comparability of assessment methods if there is more than one group	7–10
Bias	9	Describe any efforts to address potential sources of bias	17
Study size	10	Explain how the study size was arrived at	7–8
Quantitative variables	11	Explain how quantitative variables were handled in the analyses. If applicable, describe which groupings were chosen and why	
Statistical methods	12	(a) Describe all statistical methods, including those used to control for confounding	9–10
		(b) Describe any methods used to examine subgroups and interactions	9–10
		(c) Explain how missing data were addressed	9–10
		(d) If applicable, describe analytical methods taking account of sampling strategy	9–10
		(e) Describe any sensitivity analyses	9–10
Results			
Participants	13	(a) Report numbers of individuals at each stage of study—e.g., numbers potentially eligible, examined for eligibility, confirmed eligible, included in the study, completing follow‐up, and analysed	11
		(b) Give reasons for non‐participation at each stage	11
		(c) Consider use of a flow diagram	
Descriptive data	14	(a) Give characteristics of study participants (e.g., demographic, clinical, social) and information on exposures and potential confounders	11–13 Tables 1, 2, 3, 4, 5
		(b) Indicate number of participants with missing data for each variable of interest	11–13 Tables 1, 2, 3, 4, 5
Outcome data	15	Report numbers of outcome events or summary measures	11–13 Tables 1, 2, 3, 4, 5
Main results	16	(a) Give unadjusted estimates and, if applicable, confounder‐adjusted estimates and their precision (e.g., 95% confidence interval). Make clear which confounders were adjusted for and why they were included	11–13 Tables 1, 2, 3, 4, 5
		(b) Report category boundaries when continuous variables were categorised	11–13 Tables 1, 2, 3, 4, 5
		(c) If relevant, consider translating estimates of relative risk into absolute risk for a meaningful time period	N/A
Other analyses	17	Report other analyses done—e.g., analyses of subgroups and interactions, and sensitivity analyses	11–13 Tables 1, 2, 3, 4, 5
Discussion			
Key results	18	Summarise key results with reference to study objectives	14
Limitations	19	Discuss limitations of the study, taking into account sources of potential bias or imprecision. Discuss both direction and magnitude of any potential bias	17
Interpretation	20	Give a cautious overall interpretation of results considering objectives, limitations, multiplicity of analyses, results from similar studies, and other relevant evidence	14–18
Generalisability	21	Discuss the generalisability (external validity) of the study results	14
Other information			
Funding	22	Give the source of funding and the role of the funders for the present study and, if applicable, for the original study on which the present article is based	1

## Appendix C See Table C1

**TABLE C1:** Opioid dependence treatment among a sample of people with opioid dependence in New South Wales, Australia, who are currently receiving or previously received OAT (*N* = 365).

Variable	Total (*N*)	Overall, *N* = 365		Participants currently receiving OAT (A), *N* = 312		Participants previously receiving OAT (B), *N* = 53	
		*n*	% (95% CI)	*n*	% (95% CI)	*n*	% (95% CI)
OAT medications received (lifetime)							
Methadone	359	307	84% (79%–87%)	266	85% (81%–89%)	41	77% (63%–87%)
Sublingual buprenorphine/buprenorphine‐naloxone	359	236	65% (60%–70%)	200	64% (58%–69%)	36	68% (53%–79%)
Long‐acting injectable buprenorphine	359	139	38% (33%–43%)	123	39% (33%–45%)	16	30% (19%–45%)
Other opioid dependence treatments received (lifetime)							
Residential rehabilitation	365	235	64% (59%–69%)	204	65% (60%–70%)	31	59% (47%–72%)
Outpatient counselling or therapy	365	197	54% (49%–59%)	170	55% (50%–60%)	27	51% (39%–64%)
Naltrexone	365	38	10% (8%–13%)	33	11% (9%–14%)	5	9% (4%–19%)

Abbreviations: CI, confidence interval; OAT, opioid agonist treatment.
